# E4orf1 Enhances Glucose Uptake Independent of Proximal Insulin Signaling

**DOI:** 10.1371/journal.pone.0161275

**Published:** 2016-08-18

**Authors:** Ha-Na Na, Vijay Hegde, Olga Dubuisson, Nikhil V. Dhurandhar

**Affiliations:** Infection and Obesity Laboratory, Pennington Biomedical Research Center, Louisiana State University System, Baton Rouge, Louisiana, United States of America; Tohoku University, JAPAN

## Abstract

Impaired proximal insulin signaling is often present in diabetes. Hence, approaches to enhance glucose disposal independent of proximal insulin signaling are desirable. Evidence indicates that Adenovirus-derived E4orf1 protein may offer such an approach. This study determined if E4orf1 improves insulin sensitivity and downregulates proximal insulin signaling in vivo and enhances cellular glucose uptake independent of proximal insulin signaling in vitro. High fat fed mice were injected with a retrovirus plasmid expressing *E4orf1*, or a null vector. E4orf1 significantly improved insulin sensitivity in response to a glucose load. Yet, their proximal insulin signaling in fat depots was impaired, as indicated by reduced tyrosine phosphorylation of insulin receptor (IR), and significantly increased abundance of ectonucleotide pyrophosphatase/phosphodiesterase-1 (ENPP1). In 3T3-L1 pre-adipocytes E4orf1 expression impaired proximal insulin signaling. Whereas, treatment with rosiglitazone reduced ENPP1 abundance. Unaffected by IR-KD (insulin receptor knockdown) with siRNA, E4orf1 significantly up-regulated distal insulin signaling pathway and enhanced cellular glucose uptake. In vivo, E4orf1 impairs proximal insulin signaling in fat depots yet improves glycemic control. This is probably explained by the ability of E4orf1 to promote cellular glucose uptake independent of proximal insulin signaling. E4orf1 may provide a therapeutic template to enhance glucose disposal in the presence of impaired proximal insulin signaling.

## Introduction

Insulin binds to the alpha (α) subunit of IR in the cell membrane, and triggers the tyrosine kinase activity in the β subunit of IR by phosphorylation of the substrates (IRS1 and IRS2) [1, 2]. This constitutes the ‘proximal’ insulin signaling, which is followed by ‘distal’ insulin signaling that involves activation of the Ras-MAPK pathway and the activation of phosphatidylinositol-3-kinase (PI3K). The activation of PI3K pathway leads to Akt activation and glucose transporter (GLUT)-4 mediated glucose uptake [3, 4]. Among other modulators of insulin signaling, Ectonucleotide pyrophosphatase/phosphodiesterase 1 (ENPP1) is a transmembrane glycoprotein expressed in adipocytes and also found in other tissues involved in glucose and lipid metabolism. ENPP1 specifically interacts with the α subunit of IR [5], leading to decreased insulin-mediated activation of phosphorylation in IR and downstream insulin signaling activation. Hence, a reduced ENPP1 expression is an indicator of efficient insulin signaling [6].

In obesity or Type 2 diabetes, cellular glucose uptake is often suboptimal due to impaired proximal insulin signaling including IR, IRS and ENPP1 [7–10]. Yet, many anti-diabetic drugs attempt to rely on a functional insulin signaling pathway to improve glucose clearance. Instead, therapeutic agents that improve glucose disposal independent of the impaired proximal insulin signaling may be highly desirable. The E4orf1 protein derived from human adenovirus Ad36 offers such a promising option.

In vitro, Ad36 increases glucose uptake in pre-adipocytes, adipocytes, and skeletal muscle cells independent of insulin, via its E4orf1 protein (E4 open reading frame 1) [11–15]. In vivo, Ad36 or E4orf1 improve high-fat diet induced hyperglycemia and up-regulate the distal insulin signaling in muscle, and adipose tissue for glucose clearance [14–17]. Via E4orf1, Ad36 activates PI3K and up-regulates Ras, and Akt signaling leading to GLUT4 translocation and abundance, which likely enhances cellular glucose uptake [12, 14, 18–22]. This property of E4orf1 could be harnessed to develop an anti-hyperglycemic agent that operates independent of proximal insulin signaling.

Adipose tissue is a key participant in E4orf1-induced improvement in glucose disposal as the expression of E4orf1 in adipocytes was sufficient to improve glycemic control in mice [17]. In response to a glucose load, adipocyte-specific transgenic E4orf1 expression in these mice significantly lowers insulin response, yet significantly improves glucose excursion, compared to control [17]. The subdued insulin response indicated that its requirement to promote glucose disposal is reduced. This may either be due to increased tissue sensitivity to a given amount of insulin, or the ability of E4orf1 to enhance cellular glucose uptake independent of insulin, resulting in reduced need for insulin secretion for glucose clearance.

To test some fundamental elements of this novel concept, separate experiments determined if E4orf1 1) appears to improve sensitivity of endogenous insulin in mice, 2) downregulates proximal insulin signaling in vivo, and 3) enhances cellular glucose uptake independent of proximal insulin signaling.

## Materials and Methods

Experimental outlines are described below. Details of assays are presented under “Techniques and assays” (T&A) section.

### Animal Experiments

Institutional Animal Care and Use Committee (IACUC) of the Pennington Biomedical Research Center approved the protocols for animal studies. Mice were purchased from The Jackson Laboratories (Bar Harbour, Maine, USA) and placed on a 12h light-dark cycle at 25°C and housed in micro-isolator cages under Biosafety level-2 containment, with ad libitum access to food and water. End of study sacrifice was conducted by CO_2_ asphyxiation followed by cervical dislocation.

### Experiment 1: Does E4orf1 appears to improve sensitivity of endogenous insulin

Nine week old C57Bl/6 male mice on HF diet (60% kcal; Research Diets Inc. D12492i) since 6 week of age were weight matched, divided into two groups and inoculated with 300μL pBabe-puro (Control) or pBabe-*E4orf1* (10^8^ copies of *E4orf1*) retrovirus as a combination of intra-peritoneal (i.p.), intra muscular (I.M.) and subcutaneous injections. Glucose tolerance test (GTT) was performed 1 week post infection (p.i.). Mice were re-inoculated 2 week p.i. Blood glucose and serum insulin was determined over time (0, 15, 30, 60 and 120 min post a bolus of 1.5 g/kg glucose) 7 days post re-inoculation. Insulin sensitivity was measured as a product of glucose and insulin [23, 24].

### Experiment 2: Does E4orf1 downregulate proximal insulin signaling in adipose tissue

Thirteen week old C57BL/6 mice on high fat diet (HFD; 60% kcal) since 6 weeks of age and inoculated with retrovirus expressing pBabe-*E4orf1* (n = 3) and pBabe-puro (Control; n = 3) were sacrificed by CO_2_ asphyxiation and cervical dislocation. GTT on these mice were reported earlier[16]. Inguinal, epididymal, and retroperitoneal fat depots were carefully separated, weighed, flash frozen in liquid nitrogen, and stored at -80°C. Protein lysates from tissues were immunoprecipitated with anti-IR antibody and immunoblotted with pIR (Try1322) antibody. Protein lysates were also used for western blot analysis to determine changes in protein expression of ENPP1 in response to E4orf1 expression as described in T & A.

### Experiment 3: E4orf1 enhances cellular glucose uptake independent of proximal insulin signaling

a) Murine 3T3-L1 pre-adipocytes were infected with pBabe retrovirus expressing *E4orf1* or a null vector, or treated with 10 nM rosiglitazone. Following 48 h infection or rosiglitazone treatment, the cells were treated with 100 nM insulin for 30 mins, lysed in RIPA buffer and cell lysates collected as described in T & A. Cell lysates were separated by SDS-PAGE and subjected to western blotting to detect protein bands as described in T & A.

b) 3T3-L1 pre-adipocytes stably transfected with inducible *E4orf1* or pTRE null vector were exposed to doxycycline (1000 ng/mL) for 24 h to induce *E4orf1* expression. Following E4orf1 induction, cells were lysed and protein extracts from cells immunoprecipitated with anti-IR and anti-IRS1 antibody as described in T & A. The immunoprecipitated proteins were separated on a SDS-PAGE gel and immunoblotted with pIR (Tyr1322) and pIRS1 (S332) antibody.

c) 3T3-L1 pre-adipocytes stably transfected with inducible *E4orf1* or pTRE null vector were transfected with small interfering RNA (siRNA) against IR, or with non-targeting (NT) siRNA, and 24-h post transfection, the cells were induced with doxycycline (1000 ng/mL) for 24 h. Following E4orf1 induction glucose uptake assay was performed to determine cellular glucose uptake by these cells under basal and insulin stimulated conditions as described in T & A.

d) To determine if the observed glucose uptake in Experiment 3 (c) was due to translocation of glucose transporter4 (Glut4) from cytoplasm to the membrane, in a parallel experiment we collected 3T3-L1 pre-adipocytes expressing *E4orf1* or pTRE null vector cells transfected with siRNA against IR and NT siRNA and performed flow cytometry as described in T & A.

e) In a parallel experiment similar to Experiment3 (c), protein lysates were extracted from E4orf1 induced cells transfected with siRNA against IR and NT siRNA to determine if E4orf1 increased cellular glucose uptake by upregulating the Ras/PI3K distal insulin signaling pathway under conditions of impaired IR signaling. Protein lysates from cells were immunoblotted with IR, Ras, AKT, AKT1, AKT2 and their phospho-form antibodies as described in T & A.

## Technique and Assays

### Retrovirus

The human adenovirus serotype 36 (Ad36) early gene 4 open reading frame 1 (E4orf1) gene was amplified by PCR and cloned into the pBabe-puro retroviral vector (Cell Biolabs, Inc) at the BamHI-EcoRI site using restriction enzyme analysis. The constructed pBabe-E4orf1 and control pBabe-puro vectors were transformed into E. coli to make DNA stocks. The DNA was transfected into BOSC-23 cells provided by Dr. E. Floyd (Pennington Biomedical, Baton Rouge, LA) to generate viral stocks and stored at -80°C. Biosafety level 2 safety measures were used during the viral stock generation and handling.

### Glucose tolerance test

Subsequent to a 4-h fast, conscious mice were injected with D-glucose (1.5 mg/kg of body weight) intra-peritoneally. Blood was collected from the tail vein prior to glucose injection (time 0) and at 15, 30, 60, and 120 min post-injection. Blood glucose was determined using a glucometer (Breeze contour, Bayer).

### Serum insulin

As described in glucose tolerance test, blood from tail vein was collected pre and post glucose injection during the indicated time points. Serum was separated by centrifugation at 5000 rpm for 20 min and collected. Serum insulin was determined using a microtiter plate assay (Rat/Mouse Insulin ELISA, #EZRMI-13K, Millipore) as per manufacturer’s instructions and the plates were read at 450 nm and 590 nm absorbance on a plate reader.

### Cell culture and Transfection

3T3-L1 fibroblast cells obtained from ATCC (#CCL-92-1), were maintained in Dulbecco’s modified eagle medium (#10-017-CV; Cellgro Inc.) with 10% normal calf serum (#SH30072.03; Hyclone) and 1% antibiotics (#A5955; Sigma Aldrich). 3T3-L1 preadipocytes were plated in 6-well plates and infected with 200 μl of pBabe-puro or -E4orf1 retrovirus per well (10^8^ copies of E4orf1) or treated with 10 nM rosiglitazone (R2408; Sigma Aldrich) for 48 h. Cells were also stimulated with 100 nM insulin (I6634; Sigma Aldrich) for 30 min. In another experiment, 3T3-L1 preadipocytes were developed for stable expression of E4orf1 using a TetOn system, pTRE-TIGHT (Null vector control; pTRE) or pTRE-E4orf1-TIGHT (E4orf1). The pTRE and E4orf1 cells were plated in 6 well plates, and transfected with 200 nM of insulin receptor (IR) siRNA or non-targeting (NT) siRNA using lipofectamine^2000^ (5 μL per well; #11668019; Life technology). Following transfection for 24 h, media was replaced with media containing 1 μg/mL doxycycline (#631311; Clonetech) and cells incubated for a further 24 h to induce E4orf1 expression. These cells were also stimulated with 100 nM insulin (I6634; Sigma Aldrich) for 30 min to determine glucose uptake.

### Immunoblotting

Cultured cells and mice tissue samples were lysed in RIPA buffer (#sc-24948; SantaCruz Biotechnology) with added protein inhibitor cocktail. Protein from the lysates was collected after centrifugation (13,000 G, 4°C, 15 min) and measured by bicinchoninic acid protein assay (#B9643, #C2284; Sigma aldrich). 30 μg lysates were subjected to SDS-PAGE (7.5 or 15% polyacrylamide), and transferred to polyvinylidene difluoride membranes (#162–0177; Bio-rad), incubated with primary antibody (IR; millipore (1:1000), p-Akt1, p-Akt2, p-Akt, t-Akt1, t-Akt2, t-Akt, Ras; cell signaling (1:1000), ENPP1; cell signaling (1:500), and then quantified by secondary antibody conjugated with horseradish peroxidase and ECL detection reagents (Amersham, #RPN2209). Protein expression was normalized to GAPDH and total AKT, and measured by densitometry using Image J software (National Institute of Health).

### Immuno-precipitation

300 μg protein lysate was incubated with anti-IR (#05–1104; Millipore) or–IRS1 (#2382; Cell signaling) monoclonal antibody (3 μg/mL) for 3 h and immuno-precipitated with the IP Matrix provided (#sc-45042, sc-45043; Santa Cruz biotechnology) overnight at 4°C. The immuno-precipitated matrix was washed 4 times with RIPA lysis buffer and boiled for 5 min with 2X sample buffer. The protein lysates were separated by SDS-PAGE electrophoresis, transferred on to PVDF membrane and immuno-blotted with p-IR (Tyr1322; #04–300; Millipore; 1:500) and p-IRS1 (Ser332/336; #2580; Cell signaling; 1:1000). The expression for phospho-proteins was normalized to anti-total IR and–IRS1, respectively.

### Flow cytometer

pTRE and E4orf1 clone cells were rinsed with phosphate buffer saline (PBS), and centrifuged at 1,500 rpm, 4°C, for 5 min, and supernatant aspirated. The cells were stained with Glut4 antibody (#ab65267, 1:50, abCam) at 4°C for 30 min, and washed twice with PBS. The cells were then stained with anti-mouse Alexa488 antibody (#A11001, 1:50, Invitrogen) at 4°C for 30 min, washed twice, and fixed with 2% paraformaldehyde. Cells were analyzed on FACS Calibur flow cytometer (Becton Dickinson). The cytometer settings for both forward scatters (FSC) and side scatters (SSC) to analyze cells were dependent on the analytic sensitivity of this machine. Therefore voltages between scatters were set to a degree so the majority of control cells (pTRE cell; null-vector control; non-treated cells), were located between the scale of 200 and 800 for the FSC and between the scales of 100 and 300 for the SSC. The relative expression of Alexa488 flour-secondary antibody was checked based on the separation of Glut4 positive cells. Data was analyzed using CellQuest Pro (Becton Dickinson) software.

### Glucose uptake

Cells were exposed to serum free media for 2 h, then washed twice with PBS, followed by 112.5 μL Krebs-Ringer phosphate buffer treatment (136 mM NaCl, 4.7 mM KCl, 10 mM NaPO_4_, 0.9 mM CaCl_2_, 0.9mM MgSO_4_). To determine non-specific glucose uptake, one well of cells was treated with100 nM cytochalasin B (#6762, Sigma Aldrich). Next, 12.5 μL of 10X isotope solution was added to each well to a final concentration of 100nM 2-deoxyglucose and 0.5 μCi ml-1 [3H]-2-deoxyglucose (#NEC720A250UC, PerkinElmer, Waltham, MA, USA) for 5 min. Cells were immediately washed in ice-cold PBS. Next, 500 μL of 0.05% SDS was added to each well, and incubated at 37°C for 30 min. 450 μL of cell lysate from each well was added to individual scintillation vials. The remaining 50 μL cell lysate was used for protein determination with BCA assay. The scintillation counts per minute were normalized to protein content of each well.

### Statistics

All values are expressed as mean ± standard deviation (SD). The glucose uptake and signaling experiments were repeated three times or more, and similar results were obtained. The experiment for Glut4 translocation was repeated two times with statistically significant result each time. One-way ANOVA was used for multiple comparisons followed by tukey’s test. Groups not sharing a letter denote statistical significance. Significance was considered at *P* < 0.05.

## Results

### Experiment 1: E4orf1 appears to improve sensitivity of endogenous insulin

**Fig 1:**
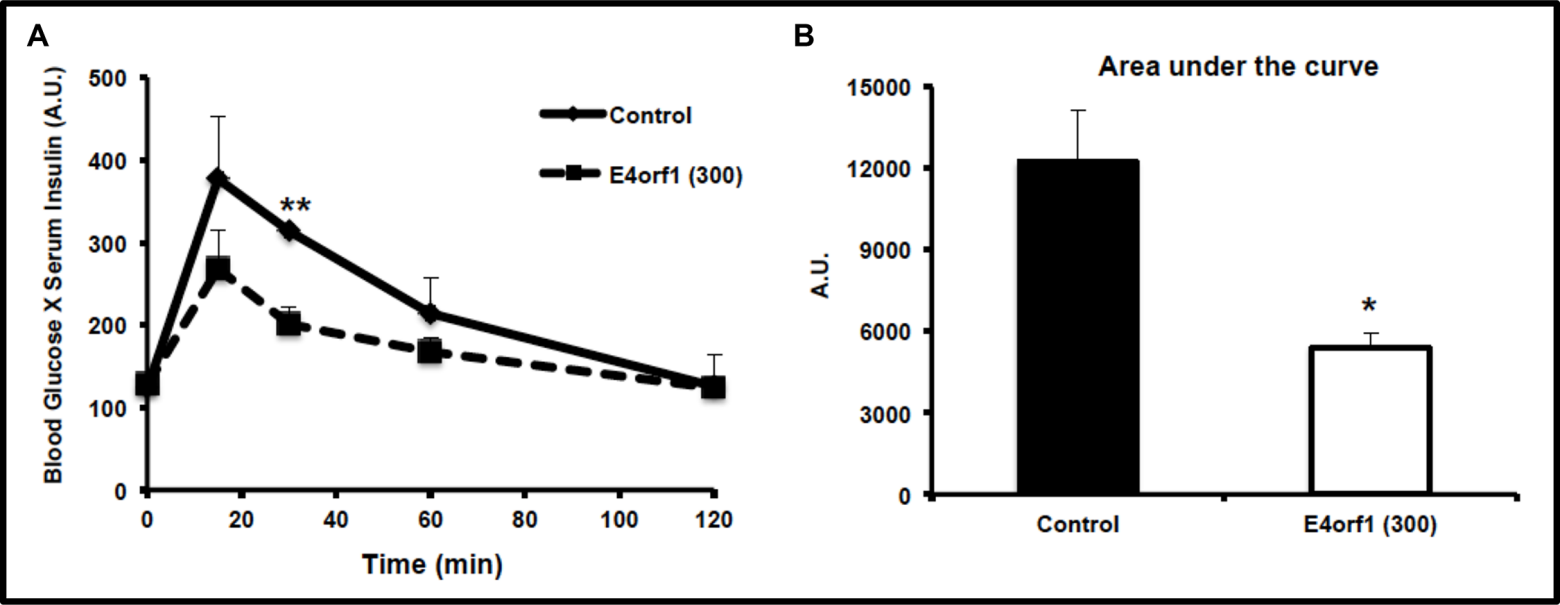
**E4orf1 expression enhances insulin sensitivity of endogenous insulin: (A)** C57BL/6J (9 week old) mice on 60% fat diet since 6 week of age were inoculated with pBabe-puro (null vector) retrovirus (control; black solid lines, n = 3), or with pBabe expressing *E4orf1* (black dotted lines; n = 3) followed by a booster inoculation one week later. Following the booster inoculation, the E4orf1 group showed greater insulin sensitivity as determined by a product of blood glucose and serum insulin in response to a glucose load (**p<0.007). Also expressed as area under the curve (AUC) **(B)**. E4orf1 expressing mice exhibit significantly smaller AUC (p<0.02).

Glucose excursion was determined by GTT in nine-week old mice on a 60% (kcal) fat diet inoculated with E4orf1 expressing retrovirus or control group of mice (data not shown). Respective groups of these mice were re-inoculated with the null vector or *E4orf1* expressing vector 7 d following the GTT. One week post re-infection, the E4orf1 group, but not the control group showed greater insulin sensitivity, measured as a product of blood glucose and serum insulin (Fig 1A). The area under curve (AUC) for E4orf1 group of mice was significantly smaller (p< 0.02) compared to control mice (Fig 1B)

### Experiment 2: E4orf1 downregulates proximal insulin signaling in adipose tissue

**Fig 2:**
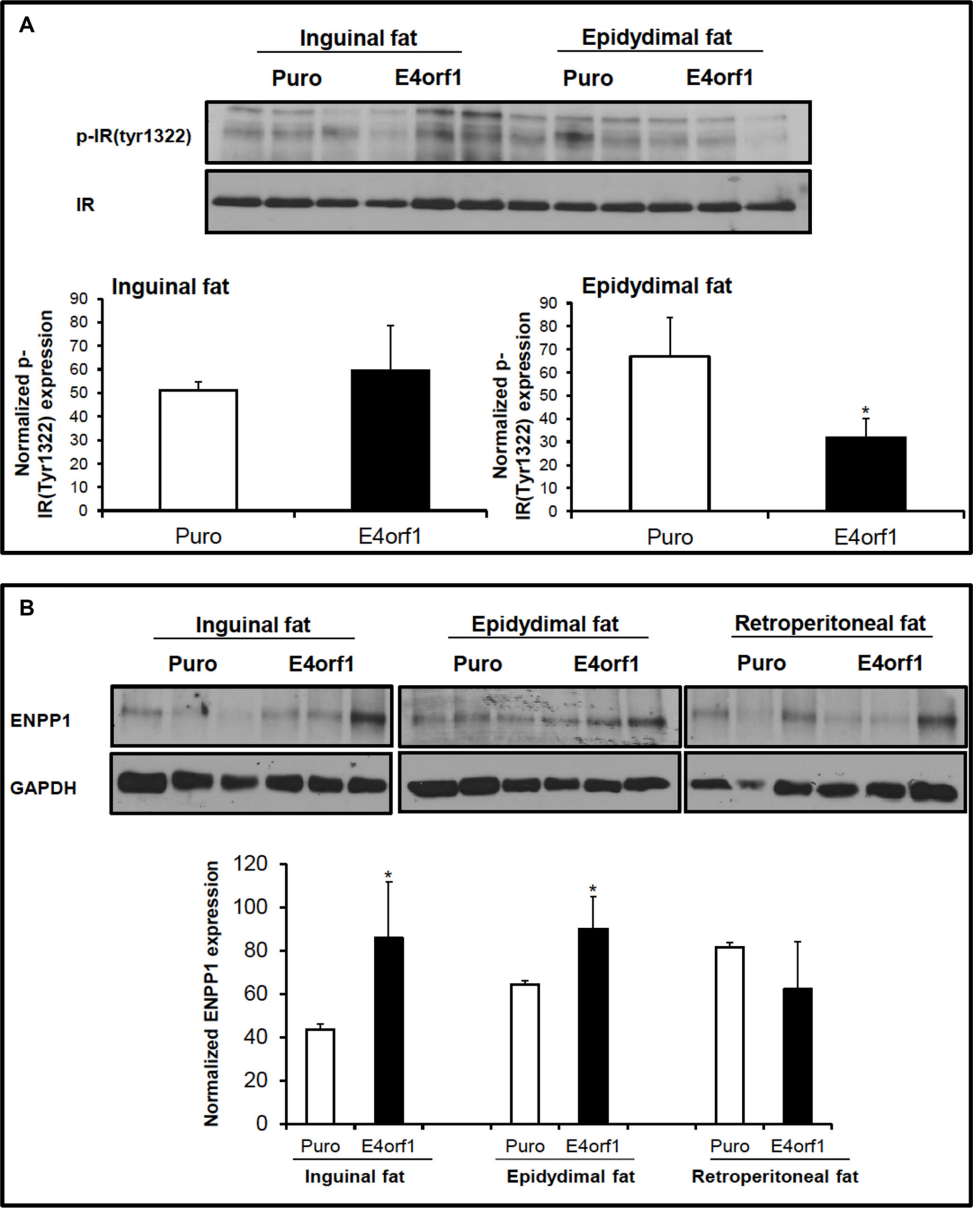
**E4orf1 downregulates proximal insulin signaling in adipose tissue: (A)** Proteins from inguinal and epidydimal fat depots of C57BL/6j mice expressing *E4orf1* or control vector were immuno-precipitated with anti-IR, and immunoblotted with p-IR (Tyr1322). **(B)** Transmembrane glycoprotein ENPP1 expression was also determined in proteins of adipose tissue depots from mice infected with pBabe-puro or pBabe-E4orf1 retrovirus using an anti-ENPP1 antibody. Mean ± SD. Data represent scanning densitometry of three western blots. (*P < 0.05 vs. Puro).

Thirteen week old mice inoculated with vector expressing *E4orf1* exhibited better glucose clearance compared to those inoculated with null vector as previously reported [16]. Here we determined insulin signaling in fat depots of these mice. Despite better glucose clearance in these HF fed mice, E4orf1 expression had impaired proximal insulin signaling in epidydimal fat depot as observed by the reduced phospho-tyrosine expression of IR (Fig 2A). No change in phospho-tyrosine expression was observed in the inguinal fat depot. E4orf1 expression significantly increased ENPP1 protein abundance in inguinal and epidydimal fat depots compared to control mice but not in retroperitoneal fat depots (Fig 2B).

### Experiment 3: E4orf1 expression enhanced cellular glucose uptake independent of proximal insulin signaling

Markers of proximal insulin signaling impairment by E4orf1 expression were further determined in vitro.

**Fig 3:**
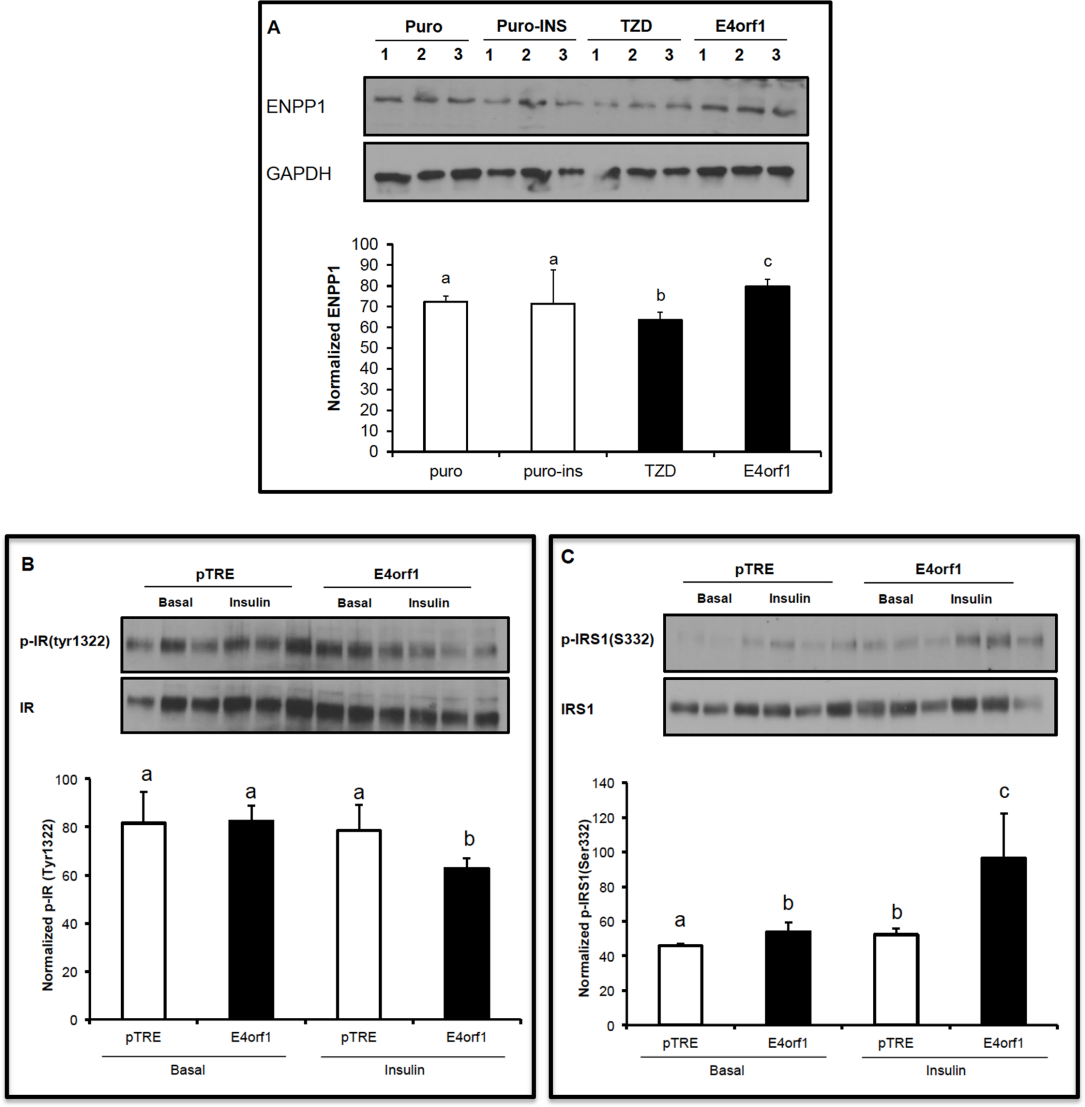
E4orf1 downregulates components of proximal insulin signaling in 3T3-L1 cells. **(A)** 3T3-L1 preadipocytes were infected with pBabe-puro or pBabe-E4orf1 retrovirus, and also treated with rosiglitazone. The puro-ins group was treated with insulin for 30 min. ENPP1 expression was evaluated following SDS-PAGE electrophoresis and immunoblotting with an anti-ENPP1 antibody. pTRE and *E4orf1* expressing 3T3-L1 cells were exposed to doxycycline to induce E4orf1 expression. Cellular proteins were immuno-precipitated with anti-IR antibody or anti-insulin receptor substrate (IRS1), and then immunoblotted with **(B)** phosphorylated IR (Tyr1322) or **(C)** IRS1 (Ser332/336). Means ± SD. Data represent densitometry of three western blots. Groups were compared using ANOVA followed by tukey’s test. Groups not sharing a letter denote statistical significance.

**Fig 4:**
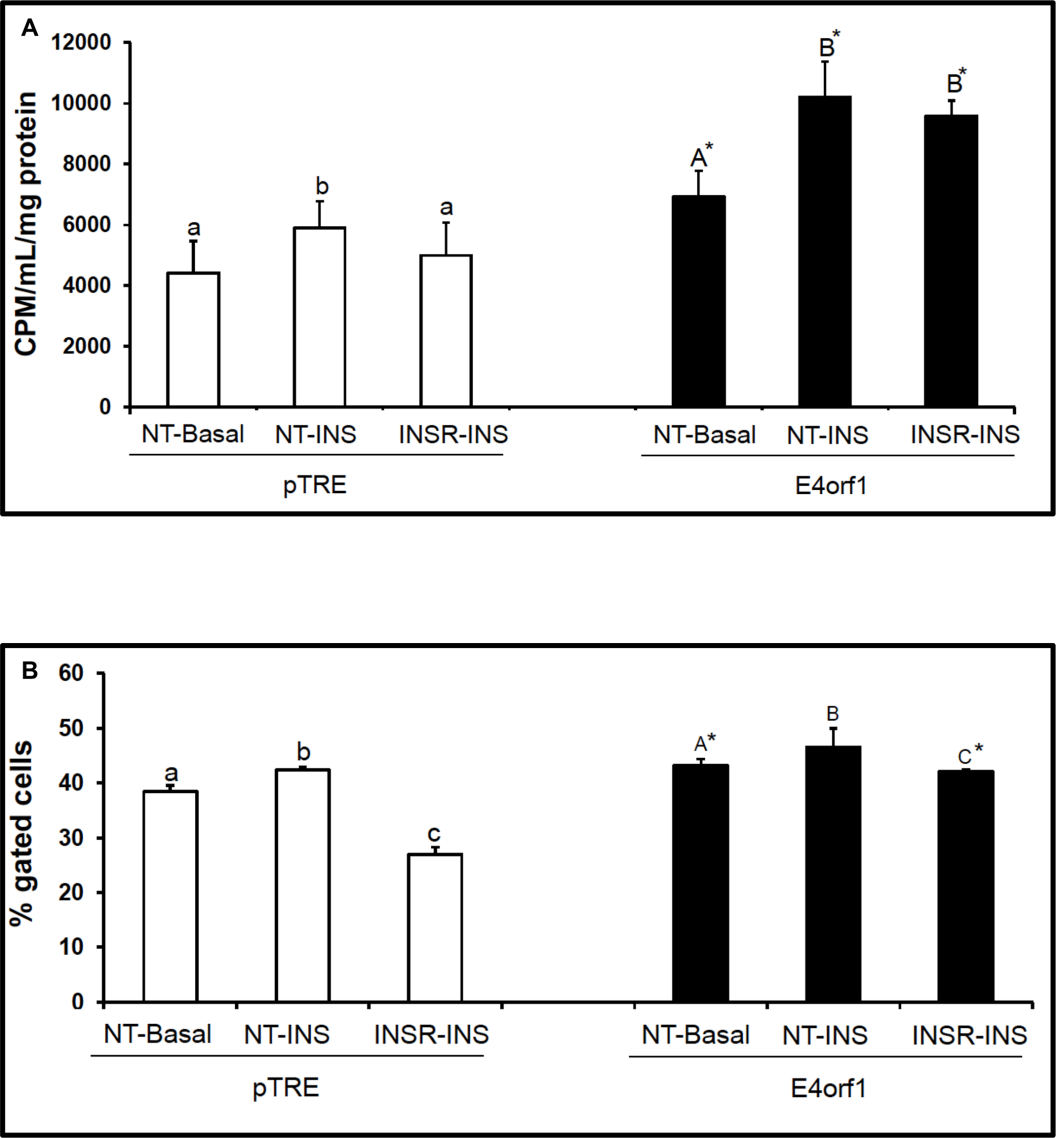
**(A) E4orf1 expression increases glucose uptake by 3T3-L1 preadipocytes despite IR knockdown.** pTRE and *E4orf1* expressing 3T3-L1 cells were transfected with IR siRNA for 6 h, followed by doxycycline treatment to induce E4orf1 expression. Glucose uptake was determined after 24 h. The experiment was repeated three times with statistically significant results. The groups were compared using ANOVA followed by tukey’s test. Groups not sharing a letter denote statistical significance. (* *P* < 0.05 vs. pTRE, NT; non-targeting, INSR; IR siRNA, Basal; non-treatment, INS; insulin treatment). **(B). E4orf1 expression in 3T3-L1 cells increases the translocation of glucose transporter4 (Glut4) even in the presence of IR knockdown.** pTRE and *E4orf1* expressing 3T3-L1 cells were transfected with IR siRNA for 6 h, followed by doxycycline treatment to induce E4orf1 expression. Next, the cells were stained with Glut4 and anti-mouse Alexa488 antibody. The cells were counted by FACS Calibur flow cytometer (Becton Dickinson) and data analyzed using CellQuest Pro (Becton Dickinson) software. Single-color staining was indicative of Glut4 expression. Histograms showed positively stained cells for Glut4. This experiment was repeated two times with statistically significant results. Groups were compared using ANOVA followed by tukey’s test. Groups not sharing a letter denote statistical significance. (* *P* < 0.05 vs. pTRE, NT; non-targeting, INSR; IR siRNA, Basal; non-treatment, INS; insulin treatment).

**Fig 5:**
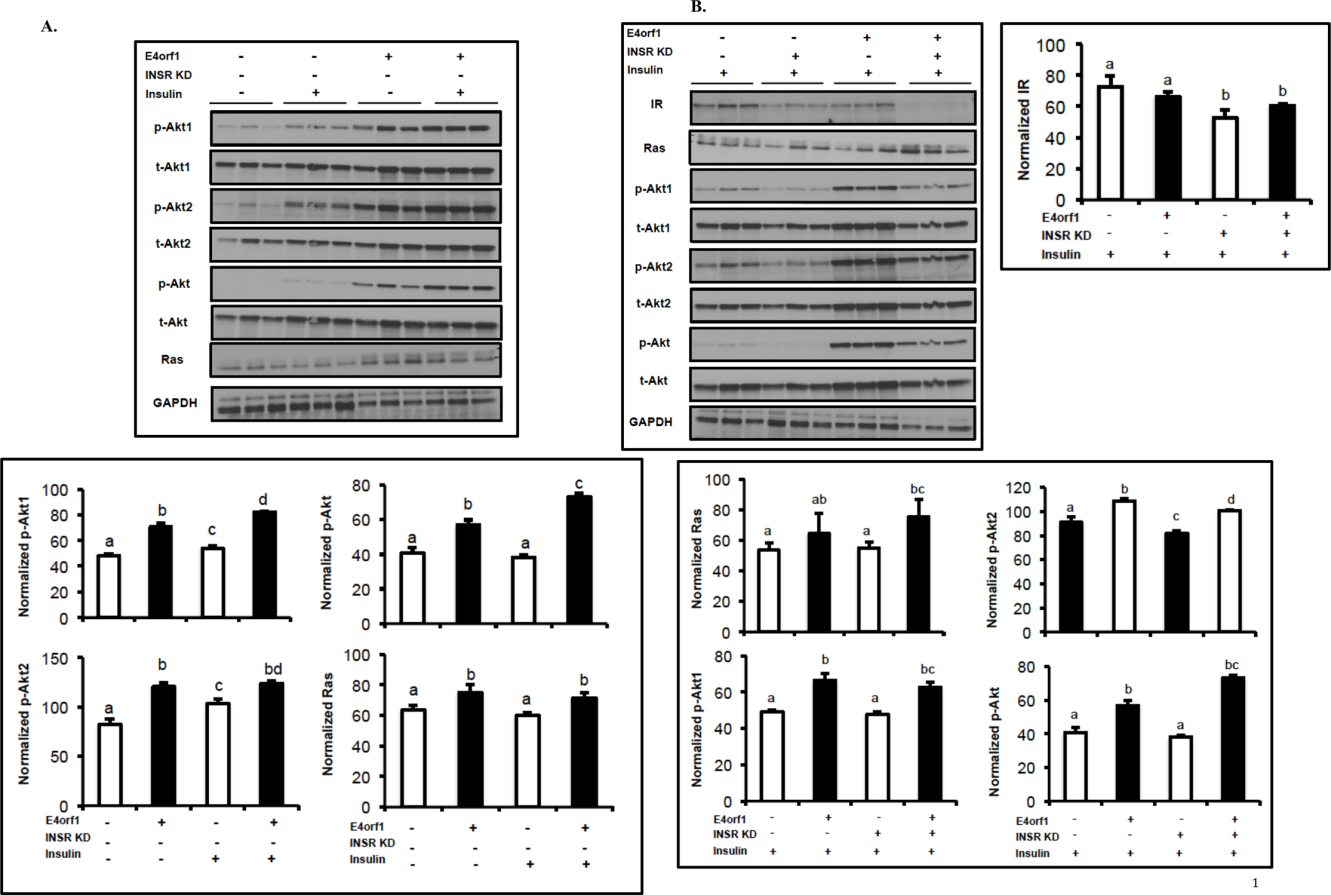
Molecular signaling of proteins in the distal insulin Ras/PI3K pathway. **(A)** pTRE and *E4orf1* expressing 3T3-L1 cells treated doxycycline treatment to induce E4orf1 expression. Protein abundance was determined 24 h later and the expressions were normalized to GAPDH. Under basal or insulin stimulated conditions, E4orf1 significantly up-regulates protein abundance of the distal insulin signaling molecules: Ras, pAKT1, pAKT2 and total pAKT. **(B)** pTRE and *E4orf1* expressing 3T3-L1 cells were treated with doxycycline to induce E4orf1 expression, followed by transfection with IR siRNA for 6 h. Under conditions of impaired proximal insulin signaling due to IR KD, E4orf1 significantly increases the protein abundance of Ras, pAKT1, pAKT2, and total pAKT. Twenty-four h later, protein expression for phospho-Akt1, Akt2, and Akt was compared after normalizing to respective total protein expressions. Mean ± SD. Data represents scanning densiometry of three western blots. Groups were compared using ANOVA followed by tukey’s test. Groups not sharing a letter denote statistical significance (*P* < 0.05, -; non-treatment, +; treatment, INSR KD; IR siRNA, Glut4; glucose transporter4, IR; insulin receptor, p-Akt; phosphorylated Akt, t-Akt; total Akt).

1. Murine 3T3-L1 cells infected with the pBabe retrovirus expressing *E4orf1* showed significantly increased ENPP1 expression compared to null vector-infected cells (Fig 3A). Insulin treatment of the null infected cells did not increase ENPP1 protein expression. Interestingly, the anti-diabetic agent rosiglitazone significantly decreased expression of ENPP1 compared with the puro control cells (Fig 3A).
2. IR-phosphorylation in inducible *E4orf1* or pTRE-null cells was determined. Under basal conditions, tyr-phosphorylation was not different between the E4orf1 or pTRE-null cells. In presence of insulin, tyr-phosphorylation of IR was significantly lower in the E4orf1 group (Fig 3B). Under basal as well as insulin-stimulated conditions, serine phosphorylation of IR was significantly greater for the E4orf1 group compared to respective pTRE-null groups (Fig 3C).
3. In pTRE-null cells, insulin stimulation increased glucose uptake compared with basal conditions in presence of non-targeting (NT) siRNA, but not when IR was knocked down (Fig 4A). However, the expression of E4orf1 significantly increased glucose uptake over 2-fold despite IR knockdown (Fig 4A).
4. To further confirm the observed glucose uptake above, Glut4 translocation from cytoplasm to membrane was determined by flow cytometry. In pTRE-null cells, insulin stimulation significantly increased Glut4 translocation in presence of non-targeting (NT) siRNA, but not when IR was knocked down with siRNA (Fig 4B). However, E4orf1 expression in these cells significantly increased Glut4 translocation despite IR knock down (Fig 4B).
5. Compared to control cells, E4orf1 significantly up-regulated protein abundance of Ras, pAKT1, pAKT2 and total pAKT (Fig 5A) under basal or insulin stimulated conditions. This confirmed that E4orf1 up-regulates the distal insulin signaling. Next, we determined if E4orf1 up-regulated distal insulin signaling when the proximal insulin signaling was attenuated by IR knockdown. Despite IR knockdown (confirmed–Fig 5B), E4orf1 significantly increased the protein abundance of Ras, pAKT1, pAKT2, and total pAKT (Fig 5B).

## Discussion

Conceptually, these results add to our understanding of determinants of glycemic control.

Systemic blood glucose excursion in response to a given load of glucose is highly dependent on the amount of insulin and sensitivity of body tissue to insulin signaling. Higher insulin amount is needed for glucose clearance if insulin signaling is impaired. Whereas, a quicker glucose clearance in presence of lower insulin amounts is indicative of better sensitivity to insulin signaling.

E4orf1 enhances glycemic response to glucose load in mice and reduces circulating insulin levels [16, 17]. Conventionally, this would be interpreted as greater sensitivity to insulin signaling due to E4orf1, which is also supported by Experiment 1 of this study. However, reality may be more complex. Insulin tolerance test conducted using exogenous insulin did not indicate greater sensitivity to insulin signaling in presence of E4orf1[16]. Importantly, Experiments 2 and 3 of this study confirmed that E4orf1 in fact attenuates the proximal insulin signaling in vivo and in vitro, and up-regulates glucose disposal independent of insulin receptor signaling. This argues against increased sensitivity to insulin signaling in presence of E4orf1. Moreover, E4orf1 does not influence insulin production or secretion from pancreatic beta cells[16]. Hence, E4orf1 is not likely to be a sensitizer, mimetic or secretagogue of insulin. Instead, we deduce that, “E4orf1 bypasses the proximal insulin signaling, but recruits distal insulin signaling to up-regulate cellular glucose uptake. Thereby, E4orf1 reduces insulin requirement for glucose disposal, which results in lower level of insulin”. This phenomenon could be described as the “insulin sparing action” of E4orf1. Insulin serves metabolic functions other than glucose clearance. If or how these functions are affected by lower insulin levels in presence of E4orf1 is unknown.

More specifically, E4orf1 provides a template to enhance glucose disposal by bypassing the impaired proximal insulin signaling, which is common in diabetes and obesity. Experiment 2 observed that E4orf1 impairs proximal insulin signaling in some tissues, as indicated by ENPP-1 abundance and phosphorylation of IR, and Experiment 3 confirmed that E4orf1 enhanced distal insulin signaling and cellular glucose uptake despite IR knockdown. An attractive implication of insulin signaling independent action of E4orf1 is that a drug based on E4orf1 action may be potentially beneficial for improving hyperglycemia in type 1 or type 2 diabetes.

Additional work is needed to determine the suitability of E4orf1 as a template for an anti-diabetic drug. As a next step, hyperinsulinemic euglycemic clamps could quantitate the requirement of endogenous insulin for maintaining glucose levels in presence of E4orf1. Furthermore, adipose tissue, skeletal muscle and liver are the key sites for insulin action that influence systemic glycemic control. The relative roles of these tissues and their interaction with insulin in vivo in presence of E4orf1 are unclear. In vitro, E4orf1 enhances glucose uptake in cells of adipose tissue and skeletal muscle and reduces glucose release from hepatocytes [21, 25]. It is possible that the systemically enhanced glycemic control by E4orf1 is a combined result of greater glucose clearance by adipose tissue and skeletal muscle and less hepatic glucose release. Uptake of radiolabeled glucose in rodents would further clarify the individual tissue contribution to glucose disposal in presence of E4orf1. The in vivo safety of E4orf1 in improving hyperglycemia remain unknown, and an appropriate drug delivery system is required. Nonetheless, Ad36 E4orf1 offers a research opportunity to develop a new anti-diabetic agent with multiple potential advantages and conceptually advances the use of a rather unconventional source, microbial proteins, for anti-diabetic drug development.

In summary, compared to insulin resistance, insulin sensitivity can be described as lower insulin secretion but efficient insulin signaling and better glycemic control, However, our in vivo and in vitro results collectively uncovered an interesting paradigm where proximal insulin signaling is impaired, yet insulin secretion is reduced and glucose disposal is enhanced. This paradoxical situation suggests that the amount of insulin secreted in response to glucose load may not only depend on insulin signaling. E4orf1, a 125 amino acid peptide is responsible for this action. This peptide may provide a template to develop anti-diabetic drugs that function independent of proximal insulin signaling, which is often impaired in diabetes.
